# Clinicopathological features and prognostic implications of ASCL1 expression in surgically resected small cell lung cancer

**DOI:** 10.1111/1759-7714.13705

**Published:** 2020-11-15

**Authors:** Jiacong Wei, Li Liu, Yiying Guo, Jinyao Zhang, Xin Wang, Jiyan Dong, Puyuan Xing, Jianming Ying, Lin Yang, Junling Li

**Affiliations:** ^1^ Department of Pathology, National Cancer Center/National Clinical Research Center for Cancer/Cancer Hospital Chinese Academy of Medical Sciences and Peking Union Medical College Beijing China; ^2^ Department of Medical Oncology, National Cancer Center/National Clinical Research Center for Cancer/Cancer Hospital Chinese Academy of Medical Sciences and Peking Union Medical College Beijing China

**Keywords:** ASH1, NanoString, neuroendocrine tumors, small cell lung cancer (SCLC), tissue microarray

## Abstract

**Background:**

Small cell lung cancer (SCLC) is one of the most aggressive lung cancers. Treatment of SCLC has remained unchanged during the past decades. Preclinical studies have revealed ASCL1 as a transcription regulator in the neuroendocrine (NE) differentiation and carcinogenesis of SCLC. However, there are few studies on correlation of ASCL1 expression and clinicopathological factors in resected SCLCs. Here, we aimed to analyze the ASCL1 expression of SCLC and investigate its associations with clinicopathological factors and survival.

**Methods:**

A total of 247 surgically resected pure SCLC specimens were included in this retrospective study, all of which were processed using tissue microarrays for immunohistochemistry analysis of ASCL1. A total of 48 of 247 cases were tested by NanoString for mRNA expression analysis on 50 SCLC related genes. Statistical analysis was performed using R studio and SPSS software.

**Results:**

NE scores of 48 pure SCLC specimens were calculated by analyzing 50 preselected genes. A significant correlation between NE score with both ASCL1 mRNA expression and ASCL1 protein expression were observed. For the entire cohort of 247 patients, ASCL1 was highly expressed in 42.5% of pure SCLC patients according to IHC results. Significant differences were observed between ASCL1 high and low expression groups in variables including staging, lymph node metastasis, nerve invasion and overall survival.

**Conclusions:**

In limited staged pure SCLC, ASCL1 expression was positively correlated with NE signature, pTNM stage, nerve invasion and OS. ASCL1 may therefore serve as a potential biomarker to predict prognosis as well as in the selection of patients for therapies targeting ASCL1‐regulated downstream molecules.

## Graphical abstract

ASCL1 expression is positively correlated with neuroendocrine signatures, pTNM stage, nerve invasion and OS. ASCL1 may serve as a potential biomarker to predict prognosis as well as to select patients for therapies targeting ASCL1‐regulated downstream molecules.

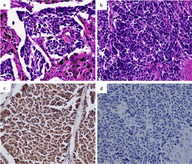

## Introduction

Small cell lung cancer (SCLC) is the most aggressive and lethal form of lung cancer, which grows fast, metastasizes early and acquires resistance in a short space of time to most current chemo and/or radiotherapies.^1^ Histologically, the majority of SCLCs manifest neuroendocrine (NE) differentiation marked by NE markers such as synaptophysin, chromogranin‐A and CD56, etc., while part of SCLCs are non‐NE subtype lacking classic NE markers.^2^, ^3^ Compared with the remarkable progresses in non‐small cell lung cancer (NSCLC), such as targeted therapies and immunotherapies, treatment of SCLC has lagged behind during the past four decades, with the standard chemotherapy regimen of platinum agents (cisplatin/carboplatin) combined with etoposide being unchanged as the first‐line treatment.^4^

A deeper understanding on the biological mechanisms of SCLC will improve refinement of the molecular subclassification scheme of SCLC and progress will be faster in therapeutic researches. Studies using cell lines, patient‐derived xenografts, genetically engineered mouse models or primary patient tumors have revealed one fundamental transcription regulator namely ASCL1, which is also known as ASH1.^5^, ^6^, ^7^ In preclinical models, ASCL1 was demonstrated as a pivot in the NE differentiation and carcinogenesis of SCLC by direct regulation of oncogenes including MYCL1, RET, SOX2, BCL2 and NFIB and a multitude of members in NOTCH signaling pathway such as DLL3.^8^, ^9^ These downstream targets of ASCL1 are “druggable” by drugs either approved in other cancer types or in clinical trials.^10^ Targeting the ASCL1 regulated pathways may serve as promising therapeutic interventions for ASCL1‐addicted SCLCs.

In several studies, the prevalence of ASCL1 expression in SCLC has been reported as 54%–73% of SCLC, which makes ASCL1 worthy of investigation as a potential therapeutic and prognostic biomarker.^11^, ^12^, ^13^, ^14^ However, validity of published data may be compromised by small cohort sizes or inclusion of combined SCLCs in which other histological types were present.^12^, ^15^ Moreover, in another study of our group to be published, we found that pure SCLC and combined SCLC have significant different expression of HIPPO pathway molecules such as YAP1. This further supports the 2015 WHO classification scheme which divide SCLC into pure SCLC and combined SCLC.^2^ In this study, we retrospectively analyzed the ASCL1 expression status of 247 surgically resected pure SCLC tumors and investigated its associations with clinicopathological factors and survival.

## Methods

### Patient selection and data collection

A retrospective study was performed using archived samples and the database at the Cancer Hospital, Chinese Academy of Medical Sciences (CHCAMS) between January 2005 and December 2016. Inclusion criteria for sample analysis was as follows: (i) histologically proven as pure SCLC without any combined histology after radical resection of lung cancer plus systemic lymph node dissection; (ii) limited stage according to The Veteran's Administration Lung Study Group's 2‐stage classification scheme (VALSG);^16^ and (iii) absence of synchronous or prior multiple primary lung cancer of other histology nor coexisting tumors from other organs. TNM stage were accessed according to the American Joint Committee on Cancer (AJCC) Cancer Staging Manual (seventh edition).^17^

Data regarding the clinicopathological characteristics, treatment history (Supplementary Table S1) and follow‐ups were also extracted from the medical records system. The study was approved by the Ethics Committee and Institutional Review Boards, and all patients were exempted an informed consent as this was an archived retrospective study.

### Pathological examination and histological reassessments

Sections from the entire cohort of 247 cases were reassessed by one senior (Yang L) and two junior clinical pathologists (Liu L, Wei JC) in our hospital according to 2015 WHO lung tumor pathology classification standard. All archival sections were retrieved and reviewed, including H&E and immunohistochemical slides on detection of NE markers namely CD56, Synapsin (Syn), Chromogranin A (CgA) and Ki‐67 to preclude poorly differentiated squamous carcinoma, and typical or nontypical carcinoids, etc.^2^ In addition, the following histopathological characteristics were also assessed, including invasion to bronchus, vessels and nerves, spread through air spaces (STAS), tumor thrombosis and metastasis to lymph node dissection in the drainage area.

### mRNA expression and NE/Non‐NE stratification by NanoString analysis

Using the NanoString Assay (NanoString technologies, Seattle, USA), we detected mRNA expression of 50 NE related genes (Supplementary Table S2) including ASCL1 in 48 samples as mentioned in a previous study.^18^ In short, the NanoString process was as follows. A total amount of 300 ng RNA was hybridized overnight following the protocol of the manufacturer. The next day, samples were loaded onto the streptavidin‐coated cartridges and analyzed on nCounter SPRINT Profiler (NanoString technologies, Seattle, USA). The raw barcode counts were background adjusted with a truncated Poisson correction using negative control spikes and normalized relative to the positive control spikes.

NE score was then calculated as previously described by Zhang *et al*.^18^ Specifically, NE score = (correl NE – correl non‐NE)/2, where correl NE (or non‐NE) is the Pearson correlation between expression of the 50 genes in the test sample and expression of these genes in the NE (or non‐NE) cell line group. This score has a range of −1 to +1 where a positive score predicts for NE while a negative score predicts for non‐NE cell types. The higher the score in absolute value, the better the prediction. SCLC were divided into NE‐high subtype with NE score > 0 and NE‐low subtype with NE score < 0. ASCL mRNA expression level was then quantified and correlation analyzed with NE Score.

### Tissue array construction and IHC status of ASCL1

Two representative tumor cores with a diameter of 1.5 mm were selected after reviewing slides of all cases to make tissue microarrays (TMA). The 247 pairs of tissue cores were distributed in seven TMA slides blocks. IHC staining was followed after serial sectioning with a thickness of 3–5 μm. During the process of generating TMA slides, 26 cores were missing partly or totally, and re‐sections of the cores were supplemented. The rabbit polyclonal antibody against human ASCL1/MASH1 (1:200; ab74065, Abcam) was pretested on sections from the rat brain (Beijing LongMaiDaS Co.,Ltd.) and a good negative and positive control was made. ASCL1 protein expression was determined by immunohistochemistry (IHC) on the TMA. All staining steps were completed on the fully automatic Roche immunohistochemical instruments (Roche Diagnostics, Shanghai, China) according to the recommended standard protocols. According to the manufacturer's scoring algorithm, intensity was scored according to a four‐tier systems: including negative (0), no staining or less than 5% staining; weakly positive (1+), 5%–25% tumor cells stained; moderately positive (2+), 25%–50% tumor cells stained; strongly positive (3+), >50% tumor cells stained. Negative quality control sections were included for quality control. For statistical analysis, negative or low expression was defined as 0 and 1+, and high expression was defined as 2+ and 3+.

### Outcomes

Overall survival (OS) was defined as the time from treatment allocation to death due to any cause. Disease‐free survival (DFS) was defined as the time from treatment allocation to recurrence, distant metastasis or death per RECIST version 1.1. The primary endpoint of the study was OS and second endpoint of the study was DFS. Follow‐up was completed to February 2019.

### Statistical analysis

Associations between ASCL1 expression and parameters including clinicopathological characteristics and IHC markers were analyzed by the χ^2^ test or Fisher's exact test. Significance level was set at two‐sided *P* < 0.05. Cox regression on OS and DFS was separately performed to show time to event distribution. All statistical analyses were performed using SPSS software (version 23.0; IBM‐SPSS, Inc., Chicago, IL, USA).

## Results

### Patient characteristics

We retrieved 247 surgically resected limited stage pure SCLC from a Chinese based single cancer center. Clinicopathological characteristics stratified by ASCL1 high and low expression were depicted in Table 1. Young patients less than 65 years accounted for the majority of the cohort with a percentage of 82%. Male and smokers also represented the major population with percentages of 71% and 64%, respectively. According to the AJCC Cancer Staging Manual (seventh edition), 78 (31.6%) patients were stage I, 68 (27.5%) were stage II, and 101 (40.9%) were stage III. All cases were followed‐up routinely, with a follow‐up duration of 0–166 months and a median follow‐up time of 48 months. By the follow‐up deadline of 28 February 2019, 120 cases (48.6%) had tumor recurrence or metastasis and 127 were disease‐free; 89 cases (36.0%) deceased, 123 were alive and 35 were lost during follow‐up. The overall median DFS was 98 months, and the median OS was not reached. one‐, three‐ and five‐year DFS and OS rates were 0.73, 0.54, 0.52 and 0.95, 0.72, 0.65, respectively.

**TABLE 1:** Clinicopathological characteristics in ASCL1 high and low expression groups

		ASCL1		
Factors	Count (%)	Low	High	*P*‐value
Gender				
Male	175 (70.85)	101	74	0.911
Female	72 (29.15)	41	31	
Age				
≤65	202 (81.78)	115	87	0.706
>65	45 (18.22)	27	18	
Smoking history				
No	89 (36.03)	52	37	0.823
Yes	158 (63.97)	90	68	
AJCC seventh stage				
I	78 (31.58)	47	31	0.017*
II	68 (27.53)	47	21	
III	101 (40.89)	48	53	
Distant metastasis				
Lymphatic metastasis				
No	212 (85.83)	124	88	0.057
Yes	35 (14.17)	18	17	
Brain metastasis				
No	204 (82.59)	119	85	0.434
Yes	43 (17.41)	23	20	
Liver metastasis				
No	227 (91.90)	131	96	0.814
Yes	20 (8.10)	11	9	
Bone metastasis				
No	227 (91.90)	134	93	0.099
Yes	20 (8.10)	8	12	
Pleural metastasis				
No	241 (97.57)	139	102	1.000
Yes	6 (2.43)	3	3	
Pathological factors				
Lymphatic metastasis				
N0	104 (42.11)	67	37	0.047*
N1	65 (26.32)	38	27	
N2	76 (30.77)	35	41	
N3	2 (0.81)	2	0	
Pleural invasion				
No	170 (68.83)	91	79	0.061
Yes	77 (31.17)	51	26	
Bronchus invasion				
No	37 (14.98)	23	14	0.533
Yes	210 (85.02)	119	91	
STAS				
No	65 (26.31)	38	27	0.00003
Yes	182 (73.68)	104	78	
Vascular invasion				
No	41 (16.60)	25	16	0.621
Yes	206 (83.40)	117	89	
Nerve invasion				
No	163 (65.99)	102	61	0.024*
Yes	84 (34.01)	40	44	
Tumor thrombosis				
No	122 (49.39)	66	56	0.287
Yes	125 (50.61)	76	49	

Note:*indicates those with p value less than 0.05.

### Positive correlation between ASCL1 mRNA expression and NE score

**FIGURE 1:**
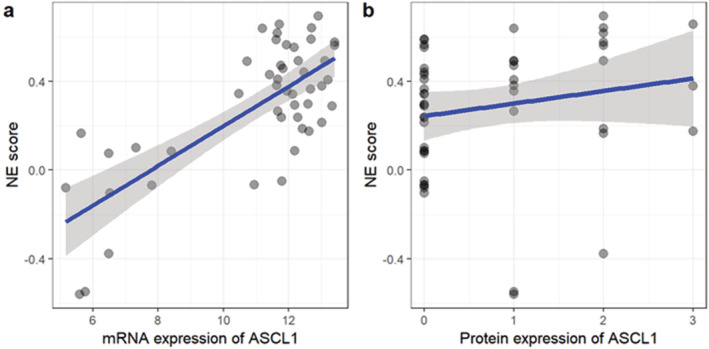
Positive correlation of ASCL1 with NE score. (a) mRNA expression of ASCL1 is positively correlated with NE score with P = 0.001 and correlation coefficient = 0.47. X‐axis represents the mRNA expression detected by NanoString. (b) Protein expression of ASCL1 is positively correlated with NE score with P = 0.044 and correlation coefficient = 0.29. X‐axis represents IHC staining of ASCL1. 0 indicates IHC negative and 1–3 indicates IHC intensity from weak to strong.

Among 247 cases, 48 cases were tested by the NanoString nCounter to access the mRNA expression of our designed panel including ASCL1. According to NE score algorithm, 40 and eight cases were stratified into NE‐high and NE‐low subtypes, respectively. ASCL1 mRNA expression averaged as 11.72 (range: 4.66–13.63) and 7.43 (range: 4.85–12.67) in the NE‐high and low groups, respectively with significant difference (*P* = 0.002). Moreover, mRNA expression of ASCL1 and NE scores were significantly correlated with each other by linear regression (*P* = 0.001) (Fig 1a, Supplementary Table S3).

### ASCL1 protein expression and its correlation with clinicopathological characteristics

**FIGURE 2:**
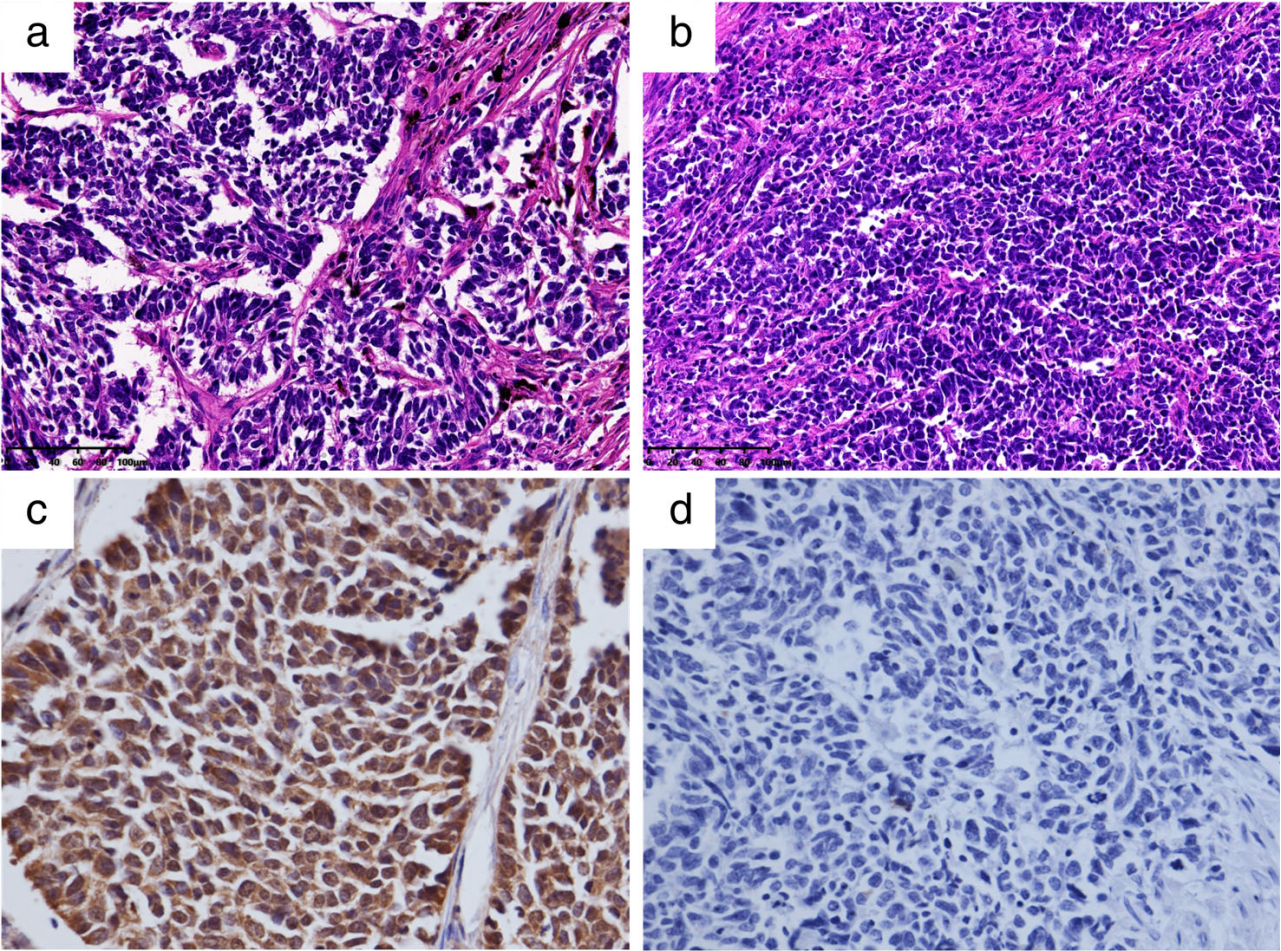
Representative H&E and ASCL1 immunohistochemical staining of pure SCLC and the corresponding IHC staining (×200). (a and c) are HE and positive ASCL1 IHC staining, respectively. (b and d) are HE and negative ASCL1 IHC staining, respectively.

ASCL1 expression is localized in the nucleus (Fig 2). For the 48 specimens tested both by NanoString for mRNA and IHC for protein, a significant correlation was observed between ASCL1 protein expression level and NE scores by Spearman's rank correlation test (*P* = 0.044, correlation coefficient = 0.29) (Fig 1b), yet we did not find significant difference of IHC intensity of ASCL1 between the NE‐high and NE‐low groups (*P* = 0.316).

For ASCL1 IHC staining of the entire cohort of 247 cases, expression of different degrees from weak to strong positivity were detected in 147 (59.5%) cases, 105 (42.5%) cases were scored as high expression (moderate and strong positivity) while 142 were low expression (no or weak positivity) according to our criteria. A significant difference by χ^2^ test between ASCL1 positive and negative groups were observed separately in TNM staging (*P* = 0.047), lymph node metastasis (*P* = 0.017), nerve invasion (*P* = 0.024) and STAS (*P* < 0.001). There was no statistical difference between ASCL1 expression levels and the rest of the clinicopathological characteristics (Table 1), nor between ASCL1 expression levels and other IHC markers including CD56, Syn, CgA, TTF‐1 and Ki‐67 (*P* > 0.05).

### Clinical outcome of patients with and without ASCL1 expression

**FIGURE 3:**
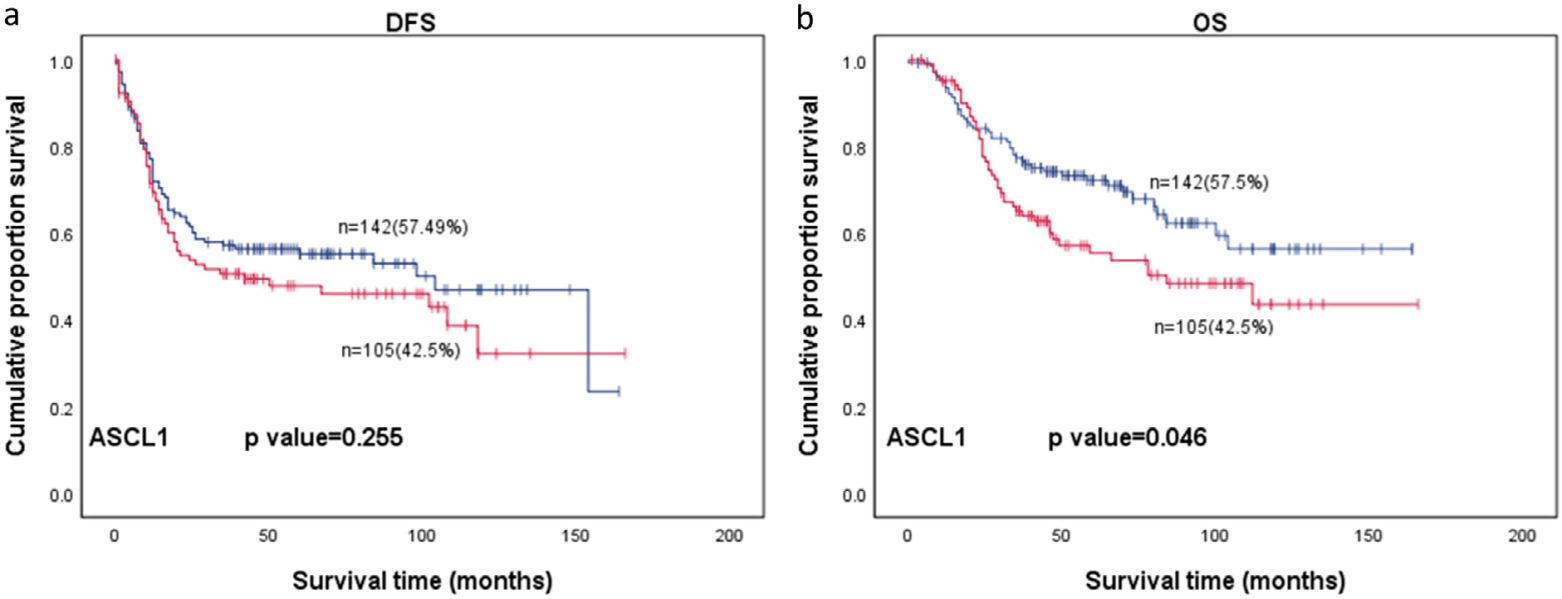
(**a**) DFS 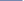, Negative; 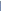, Positive; 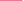, Negative‐censored; 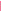, Postive‐censored and (**b**) OS of 247 patients with SCLC.

All 247 patients were included for survival analysis, with a median follow‐up time of 48 months (mean 57 months; range 0–167 months). The one‐, three‐ and five‐year DFS rates of ASCL1 lowly expressed patients were all higher than those of highly expressed patients (77% vs. 69%, 57% vs. 51%, 55*%* vs. 48%, respectively), despite being statistically insignificant (*P* = 0.255); the one‐, three‐ and five‐year OS rates of ASCL1 lowly expressed patients were higher than those of highly expressed patients (94% vs. 95%, 77% vs. 65%, 72% vs. 55%) with a significant statistical difference (*P* = 0.046) (Fig 3; Table 1). When stratifying the patients into different treatment modes, surgery followed by chemotherapy (*n* = 120) and surgery followed by chemotherapy with subsequent radiotherapy (*n* = 61), a worse prognosis trend of DFS and OS could be observed in ASCL1 highly expressed groups, although without significance (*P* > 0.05).

## Discussion

In this study, we calculated the NE scores of pure SCLC by analyzing mRNA expression of 50 preselected genes related to SCLC and specifically observed the ASCL1 gene. We found a significant correlation between NE score with ASCL1 mRNA expression and ASCL1 protein expression. ASCL1 were highly expressed in 42.5% (105/247) of pure SCLC patients, and a significant difference was found in variables including TNM staging, lymph node metastasis, nerve invasion, as well as OS. Surgery has not been recommended for SCLC since 2002 when the seventh NCCN was published.^19^ Meanwhile, it is estimated that more than 60% of patients are diagnosed with extensive stage SCLC which do not require surgical resection.^20^ Thus, studies that rely on large tissue sections are somehow hindered by the availability of SCLC tissue. Our study represents the largest cohort (*n* = 247) studying ASCL1 expression in SCLC patients with detailed treatment information and survival data. Moreover, considering the different dominating pathways between pure SCLC and combined SCLC, we only included pure SCLC in this study (*n* = 247). All patients included in the cohort were at limited stage and pure SCLC. In addition, our study included the most comprehensive clinicopathological variables for correlation analysis with ASCL1 and found a significant difference of ASCL1 expression in TNM staging (*P* = 0.017), lymphatic metastasis (*P* = 0.047) and nerve invasion (*P* = 0.024), and OS (*P* = 0.046).

ASCL1 was reported to be expressed in 54%–73% of SCLC in different studies.^11^, ^12^, ^13^, ^14^ According to the hierarchical clustering of multiple transcription regulators of SCLC, ASCL1 was expressed in 65.6% (19/29) and 73.0% (38/52) of SCLC patients without stratification by clinical stage in two studies using a Caucasian population, respectively, and 53.7% (29/54) in different cell lines from the Cancer Cell Line Encyclopedia.^11^, ^12^, ^13^, ^14^ In the study by Furuta *et al*. by defining ASCL1 positivity as nuclear staining in ≥5% of all tumor cells, the ASCL1 expression positivity rate was 64% (61/95) in surgically resected SCLC specimens in a Japanese population.^15^ In our study, we reported a high expression rate as 42.5%, which is different from the study of Megumi *et al*. This is because we defined high expression as IHC 2+ and 3+, and low expression as IHC – and 1+. If we adopted the same criteria as Megumi *et al*. our ASCL1 positive rate would be 59.5% (147/247) which is comparable. In addition, we also found that ASCL1 high expression rate varied according to TNM stage, as 40% (31/78) in stage I, 31% (21/68) in stage II and 53% (53/101) in stage III and ASCL1 expression was correlated with TNM stage in our data with a *P*‐value of 0.017 by chi square test. Thus, we suspect that patient inclusion at different TNM stages may explain the observed differences in ASCL1 positive rates.

We observed a worse prognosis tendency in ASCL1‐high patients compared with ASCL1‐low patients, where ASCL1 represents a marker for NE differentiation. Our observation is in accordance with the observation of the study by Hamanaka *et al*., in which the NE phenotype is defined by three NE markers (chromogranin A, CD56 and synaptophysin).^21^ We also observed a significant difference on OS in ASCL1 high and low expression groups, which is in accordance with the significant differences on TNM stage, lymphatic metastasis and nerve invasion. As far as we are aware, only one study from the group of Megumi *et al*. has compared the prognosis of different ASCL1 expression groups. Correlation between ASCL1 expression positivity and OS was reported to be insignificant in their cohort (*n* = 95) of both pure SCLC and combined SCLC of TNM stage I–III, in patients with only pure SCLC (*n* = 41), as well as in patients TNM stage I and II (*n* = 84, *P* = 0.139). The difference in prognosis prediction is probably due to the relatively small cohort size, the inclusion of combined SCLC, the constitution of different TNM stages or a different way to define ASCL1 groups. We admit that we neglected the influences of other factors on OS such as different surgical resection options, adjuvant or neoadjuvant chemo and/or radiotherapies, etc., which is a setback of this study.

Biologically, ASCL1 has been reported as a pivot in NE differentiation, and ASCL1 dominated SCLC shows high degree of NE differentiation.^5^, ^13^ Our data also demonstrated that both ASCL1 mRNA and protein expression are tightly correlated with NE scores which is a validated indication of NE differentiation.^18^ In addition, in preclinical studies, ASCL1 was shown to stimulate proliferation and migration in SCLC cells by targeting CDK5,^18^ which is exemplified in our data that ASCL1 high expression is significantly correlated with TNM stage, lymphatic metastasis and nerve invasion. Recently, molecular subclassification were proposed according to the four key transcription regulators names as ASCL1, NeuroD1, YAP1 and POU2F3.^2^, ^13^ We focused on ASCL1 in this study since it represents the largest constitution of the four molecular types. Clinical outcome of SCLC patients can be deteriorated by paraneoplastic syndrome caused by excessive hormone production which is associated with NE differentiation extent^.^ ^18^, ^22^ This is in accordance with our observation that ASCL1 as a pivot NE regulator is associated with poor prognosis. ASCL1 and its downstream targets such as BCL2, RET, SOX2, DLL3, NFIB and other NOTCH members can be inhibited by modulating their expression or degradation, blocking protein/protein interactions, or blocking the DNA binding of transcription regulator either through a binding pocket or at the DNA‐interacting site inhibitors.^7^, ^22^, ^23^, ^24^ ASCL1 expression, either with or without combination of other markers, may help to select patients who might benefit from drugs modulating ASCL1 signaling.

In conclusion, in the current study, a high expression of ASCL1 was found in early‐mid stage (I–III) resected SCLC, which is positively correlated with stage TNM, nerve invasion and NE signature. In future, investigation of other molecular subtypes in SCLC patients with low ASCL1 expression is of essential importance. Our study provides a convincing reference for future researches on the advent of potential targeted drugs and will allow clinicians to stratify SCLC patients into ASCL1 high and low groups according to expression level, which is of crucial importance in selecting potential patients that may benefit from treatment in the future.

## Disclosure

The authors declare that there are no conflicts of interest.

## Supporting information


**Table S1** Supporting Information

**Figure S1** Supporting Information
