# Supplementary material for: Clinicopathological features and prognostic implications of ASCL1 expression in surgically resected small cell lung cancer
Source: Thorac Cancer. 2020 Nov 15;12(1):40–7. doi: 10.1111/1759-7714.13705 (PMC7779202; doi:10.1111/1759-7714.13705)
Supplement: Supplementary file 1 — Table S1 Supporting Information [file TCA-12-40-s001.docx]

Supplementary Table 1. Summary of treatment history

| Treatment modes | Count |
| --- | --- |
| Surgery + chemotherapy | 121 |
| Surgery + chemotherapy + radiotherapy | 61 |
| Surgery with unknown follow-up treatment history | 26 |
| Chemotherapy + Surgery + chemotherapy | 12 |
| Chemotherapy + Surgery + chemotherapy+ radiotherapy | 8 |
| Surgery without chemotherapy nor radiotherapy | 5 |
| Chemotherapy + surgery | 3 |
| Surgery + concurrent chemoradiotherapy | 3 |
| Surgery + radiotherapy+ chemotherapy | 3 |
| Concurrent chemoradiotherapy + chemotherapy | 1 |
| Chemotherapy + radiotherapy+ chemotherapy | 1 |
| Chemotherapy | 1 |
| Radiotherapy+ chemotherapy + surgery | 1 |
| Surgery + chemotherapy+ radiotherapy+ chemotherapy | 1 |
| sum up | 247 |

Supplementary Table 2. 50 NE*-related genes.

| Genes with NE-High Signatures | Genes with NE-Low Signatures |
| --- | --- |
| BEX1 | RAB27B |
| ASCL1 | TGFBR2 |
| INSM1 | SLC16A5 |
| CHGA | S100A10 |
| TAGLN3 | ITGB4 |
| KIF5C | YAP1 |
| CRMP1 | LGALS3 |
| SCG3 | EPHA2 |
| SYT4 | S100A16 |
| RTN1 | PLAU |
| MYT1 | ABCC3 |
| SYP | ARHGDIB |
| KIF1A | CYR61 |
| TMSB15A | PTGES |
| SYN1 | CCND1 |
| SYT11 | IFITM2 |
| RUNDC3A | IFITM3 |
| TFF3 | AHNAK |
| CHGB | CAV2 |
| FAM57B | TACSTD2 |
| SH3GL2 | TGFBI |
| BSN | EMP1 |
| SEZ6 | CAV1 |
| TMSB15B | ANXA1 |
| CELF3 | MYOF |

Note:

NE: neuroendocrine

Supplementary Table 3. Clinicopathological features of 48 SCLC patients with NE scores.

| Characteristics | n (%) | NE-High (%) | NE-Low (%) | P-value | |
| --- | --- | --- | --- | --- | --- |
| Gender |  |  |  |  | |
| Male | 33 (68.8) | 26 (65) | 7 (87.5) | 0.406 | |
| Female | 15 (31.2) | 14 (35) | 1 (12.5) |  | |
| Age |  |  |  |  | |
| ≤60 | 32 (66.7) | 27 (67.5) | 5 (62.5) | 1.000 | |
| >60 | 16 (33.3) | 13 (32.5) | 3 (37.5) |  | |
| Smoking history |  |  |  |  | |
| Yes | 23 (47.9) | 19 (47.5) | 4 (50) | 1.000 | |
| No | 25 (52.1) | 21 (52.5) | 4 (50) |  | |
| Tumor Location |  |  |  |  | |
| Left Lung | 25 (52.1) | 20 (50) | 5 (62.5) | 0.703 | |
| Right Lung | 23 (47.9) | 20 (50) | 3 (37.5) |  | |
| AJCC 7^th^ staging |  |  |  |  | |
| I | 12 (25) | 7 (17.5) | 5 (62.5) | 0.030 | |
| II | 15 (31.2) | 13 (32.5) | 2 (25) |  | |
| III | 21 (43.8) | 20 (50) | 1 (12.5) |  | |
| Tumor size |  |  |  |  | |
| ≤3 cm | 12 (25) | 8 (20) | 4 (50) | 0.094 | |
| >3 cm | 36 (75) | 32 (80) | 4 (50) |  | |
| lymphatic metastasis |  |  |  |  | |
| Yes | 31 (64.6) | 29 (72.5) | 2 (25) | 0.017 | |
| No | 17 (35.4) | 11 (27.5) | 6 (75) |  | |
| Operation ways |  |  |  |  | |
| pulmonary lobectomy | 40 (83.3) | 34 (85) | 6 (75) | 0.309 | |
| wedge resection | 2 (4.2) | 1 (2.5) | 1 (12.5) |  | |
| radical pneumonectomy | 3 (6.2) | 2 (5) | 1 (12.5) |  | |
| NA | 3 (6.2) | 3 (7.5) | 0 (0) |  | |
| Treatment modes |  |  |  |  | |
| S+C | 25 (52.1) | 19 (47.5) | 6 (75) | 0.249 | |
| S+C+R | 23 (47.9) | 21 (52.5) | 2 (25) |  | |
| Chemotherapy regimens |  |  |  |  | |
| CE | 10 (20.8) | 9 (22.5) | 1 (12.5) | 0.257 | |
| EP | 19 (39.6) | 17 (42.5) | 2 (25) |  | |
| EL | 1 (2.1) | 0 (0) | 1 (12.5) |  | |
| CE+EP | 4 (8.3) | 3 (7.5) | 1 (12.5) |  | |
| NA | 14 (29.2) | 11 (27.5) | 3 (37.5) |  | |
| PCI |  |  |  |  | |
| Yes | 18 (37.5) | 15 (37.5) | 3 (37.5) | 1.000 | |
| No | 30 (62.5) | 25 (62.5) | 5 (62.5) |  | |
| NE: Neuroendocrine; AJCC: American Joint Committee on Cancer; S: Surgery; R: Radiotherapy; C: Chemotherapy; CE: Etoposide and carboplatin; EP: Etoposide and cis-platinum; EL: Etoposide and lobaplatin; NA: Not available; PCI: Prophylactic craniocerebral irradiation. | | | | |  |
