# Supplementary figures and images for: Clinicopathological features and prognostic implications of ASCL1 expression in surgically resected small cell lung cancer
Source: Thorac Cancer. 2020 Nov 15;12(1):40–7. doi: 10.1111/1759-7714.13705 (PMC7779202; doi:10.1111/1759-7714.13705)

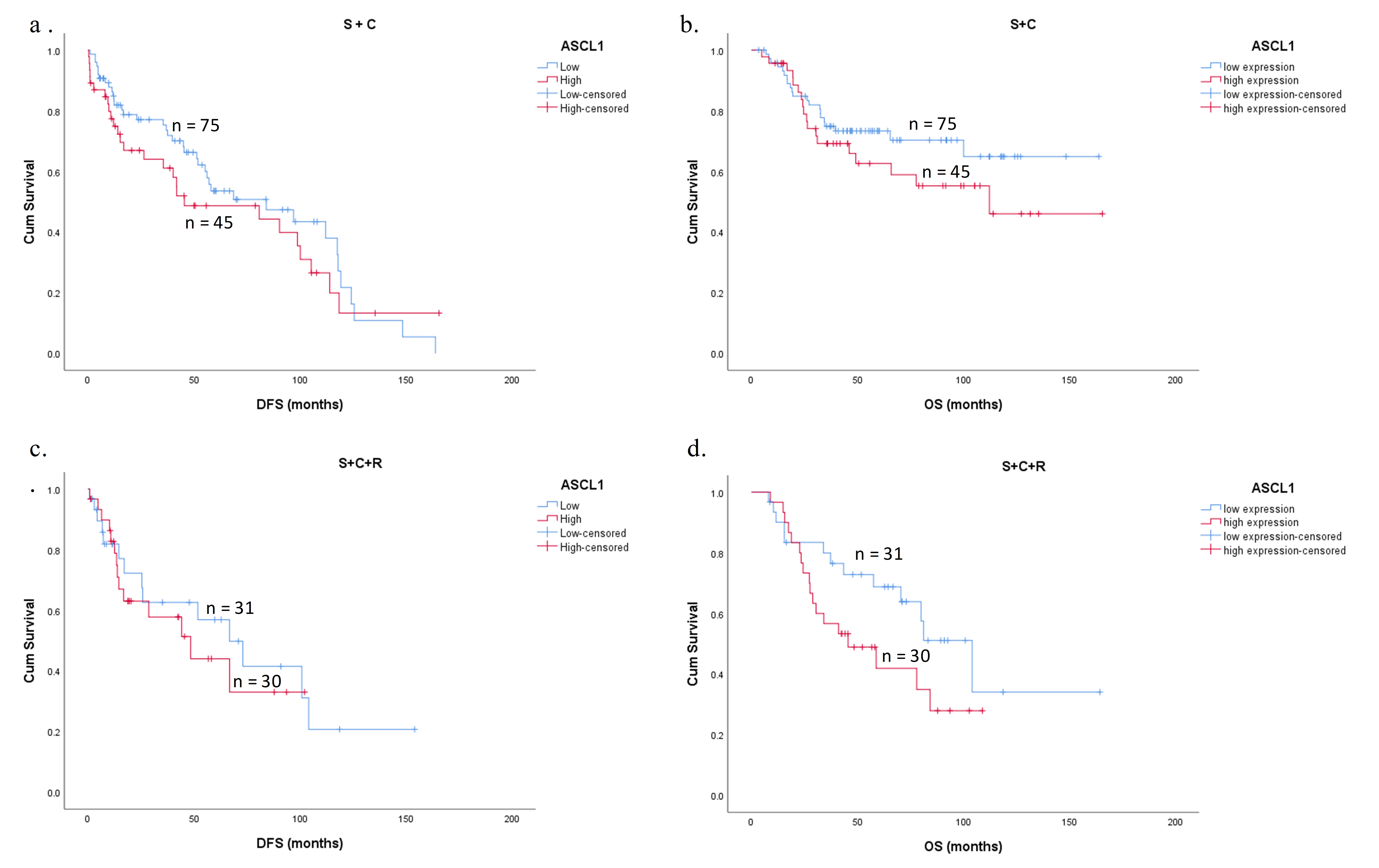

Supplement: Supplementary file 2 — Figure S1 Supporting Information [file TCA-12-40-s002.jpg]
